# Romanian Medical Students’ Attitude towards and Perceived Knowledge on COVID-19 Vaccination

**DOI:** 10.3390/vaccines9080854

**Published:** 2021-08-04

**Authors:** Ana Bălan, Ioana Bejan, Simona Bonciu, Cristina Elena Eni, Simona Ruță

**Affiliations:** 1 Faculty of Medicine, Carol Davila University of Medicine and Pharmacy, 050474 Bucharest, Romania; ioana.bejan@stud.umfcd.ro (I.B.); simona.bonciu@stud.umfcd.ro (S.B.); cristina.eni@stud.umfcd.ro (C.E.E.); simona.ruta@umfcd.ro (S.R.); 2 Stefan S. Nicolau Institute of Virology, 285 sos Mihai Bravu, 030304 Bucharest, Romania

**Keywords:** COVID-19 vaccination, medical students, SARS CoV-2 vaccine, vaccine hesitancy

## Abstract

In Romania, the first phase of the COVID-19 vaccination campaign prioritized medical personnel, which included healthcare students. This study aimed to assess their knowledge, attitudes towards, and perception of COVID-19 vaccination. An anonymous, single-answer, 42-item online survey was conducted from 12 January until 3 March 2021, in the country’s largest University of Medicine and Pharmacy. Among the 1581 respondents (14.9% response rate), 88.5% were pro-vaccination, 7.8% were undecided, and 3.7% were vaccine resistant. The main reason for vaccine rejection was the perceived speed of vaccine development (strong agreement among the vaccine resistant, moderate agreement among the undecided, *p* < 0.001). Concern over long-term adverse reaction was present in only 11.5% of the respondents, significantly more frequent in the undecided and vaccine resistant. Perceived knowledge on the vaccines’ safety, efficacy, and technology correlated with a pro-vaccine attitude (*p* < 0.001). Most respondents had a positive stance towards vaccination in general, influencing their behaviour as future parents (99.3% of the pro-vaccination, 95.1% of the undecided, and 89.1% of the vaccine resistant will vaccinate their children, *p* < 0.001) and as medical professionals (99.7% of the pro-vaccination, 93.5% of those undecided, and 89.8% of the vaccine resistant would advise parents to vaccinate their children, *p* < 0.001). Healthcare students can thus serve as important vectors for scientifically sound information, influencing vaccine uptake in the community.

## 1. Introduction

Vaccines have an indisputable impact on public health, leading to a significant decrease in the morbidity and mortality of infectious diseases, saving millions of lives annually [1]. In less than a year since the identification of the new, highly pathogenic coronavirus SARS-CoV-2, several specific vaccines became available and were deployed to a large part of the population worldwide—an important step toward the end of the pandemic. In Romania, the first case was identified on the 26 February 2020 [2], and by 21 July there were 1,081,773 confirmed cases, 34,258 deaths, and 1,046,881 recoveries. According to the National Surveillance and Control Centre for Transmissible Diseases, 14,379 cases were registered among healthcare workers (1.3% of the total number of confirmed cases) [3]. The mass vaccination campaign started on 27 December 2020, comprising three phases: the first one prioritized healthcare and social workers; the second one was for people aged over 65, chronically ill patients, and essential personnel; while the third phase was open to the entire adult population. Healthcare students from Medical School, Dentistry, Pharmacy, Nursing, and Midwifery were included in the first phase of the campaign; vaccination was offered as early as January 2021, with the first vaccine approved in the European Union—Comirnaty (produced by Pfizer/BioNTech), based on an innovative mRNA platform that encodes the SARS-CoV-2 spike protein stabilized in a prefusion conformation [4].

By 9 July 2021, 9,100,000 vaccine doses had been administered in Romania (79.1% Pfizer Biontech, 9.3% Astra Zeneca, 7.9% Moderna, and 3.7% Johnson & Johnson), with 4,640,000 fully vaccinated individuals and 170,000 receiving the first dose only [5] representing less than 40% of the eligible population in all age groups, including the most vulnerable ones (over 65 years old) [6].

Healthcare students, as future medical providers, can serve as role models in their communities. Trust in medical providers is associated with the belief that vaccines are safe, although this is less relatable for Eastern European countries [7]. Although the rapidly developed vaccines brought enthusiasm and hope in the population, a growing number of unauthorized sources, which disseminate incorrect and misleading information, have influenced the general population’s perception, leading to refusal or postponement of vaccination. This phenomenon of vaccine hesitancy is recognised as one of the top 10 threats to global health [8].

This study aimed to assess the attitude of healthcare students at the Carol Davila University of Medicine and Pharmacy in Bucharest towards the EU-approved COVID-19 vaccines, their knowledge of vaccine development, their perception on vaccine efficacy and safety, and the main reasons that stand behind vaccine hesitancy.

## 2. Materials and Methods

An anonymous, single-answer, 42-item online survey, open to healthcare students from all faculties of the Carol Davila University of Medicine and Pharmacy in Bucharest—Medicine, Dentistry, Pharmacy, Nursing, and Midwifery—was conducted using Google Forms, from 12 January until 3 March 2021. The survey was divided into 6 sections: general data, COVID-19 infection and vaccination, safety and efficacy of the EU approved COVID-19 vaccines, perceived knowledge on their development and technology, and attitude towards other vaccines. The Likert scale was used for 10 out of the 42 items, which can be found in Appendix A. The minimum required sample size was calculated to be 1343 students, using the online computer developed by the Australian Bureau of Statistics for sample size calculations, and based on a reasonable expected frequency of 50% respondents within the given population, with a confidence level of 95% (confidence interval set at +/−2.5%) [9].

For the initial descriptive analysis of the surveyed data, the tools offered by Google Forms were used to obtain a preview of the respondent population and the frequency of their chosen responses. Multiple cross-tabulations and non-parametric tests were performed using the IBM SPSS Statistics v.20 software, with a statistical significance threshold set at a *p*-value < 0.05. Since the data was mostly of nominal and ordinal type, with an expected non-gaussian distribution, the Mann-Whitney U and Kruskal-Wallis tests were employed to assess significant associations between certain questions and groups of respondents, besides the Pearson’s chi-squared test used in the cross-tabulation analysis. Respondents were split into 3 groups, according to their attitude toward COVID-19 vaccines: pro-vaccination—those that were already vaccinated at the time of the survey and those who were planning to get vaccinated; undecided—those that answered “Maybe” or “Not yet, I am waiting for my peers to get it first”; and vaccine resistant—those that answered “No”.

Answers to open-ended questions were manually and individually analysed and grouped according to several keywords frequently used in the respondents’ replies. The more complex answers, which fitted more than one category, were analysed separately in order to provide an accurate overview of the respondents’ perception.

## 3. Results

### 3.1. The Study Population

There were 1581 responders, 72% of whom are studying General Medicine, 14% Dentistry, 11% Pharmacy, and 3% Nursing and Midwifery. The 1581 respondents are a representative sample, according to the study methodology, although they account for 14.9% of the entire student population of 10,608 students in the university (14.52% of the total medical students, 16.71% of the total dentistry students, 21.95% of the total pharmacy students, and 6.98% of the total nursing and midwifery students).

Of all respondents, 74.5% are females and 87.6% come from urban areas, the majority (61.4%) are 21–25 years old, while 36.1% are 18–20 years old, and only 2.4% are >25 years old.

### 3.2. COVID-19 Infection and Vaccination

Only 11.6% of the respondents were previously diagnosed with SARS-CoV-2 infection, the majority of whom had mostly mild (52.1%) or moderate (35.8%) symptoms; the rest (12.1%) was asymptomatic, while no respondent reported a severe clinical form. Only 5.4% of the respondents had a diagnosed chronic illness.

### 3.3. Attitude towards Vaccination

Over 88% of all respondents have a positive attitude toward vaccination, with 42.5% having been vaccinated in the first month of the national campaign and 46% willing to be vaccinated. Out of the 184 respondents who have previously been infected with SARS-CoV-2, 155 (84.2%) are pro-vaccination. There were no statistically significant associations between the willingness to get vaccinated and the participants′ age, gender, urban or rural origin, previous COVID-19 infection status, and severity of disease in those previously infected or having chronic illnesses.

The undecided group represents 8% of the respondents and include those who answered “maybe” and “not yet, I am waiting for my colleagues to get it first” when asked if they are willing to get vaccinated.

The vaccine-resistant group represents only 4% of all respondents and are those who refuse to be vaccinated. Moreover, only 11 respondents (0.7%) stated that nothing would change their minds about the choice of not getting the vaccine.

As per Table 1, medical students are more likely to adopt a pro-vaccination stance compared to their peers (91.9% vs. 81.9% of those in Dentistry, 77.1% of those in Pharmacy, 77.3% of those in Nursing and Midwifery Schools, *p* < 0.001) and the least likely to be undecided (5.6% vs. 12.5% of those in Dentistry, 14.3% in Pharmacy, and 15.9% in Nursing and Midwifery, *p* < 0.001).

A total of 84.9% of all respondents believe that healthcare students have a higher risk of SARS-CoV-2 infection than the general population.

**Figure 1:**
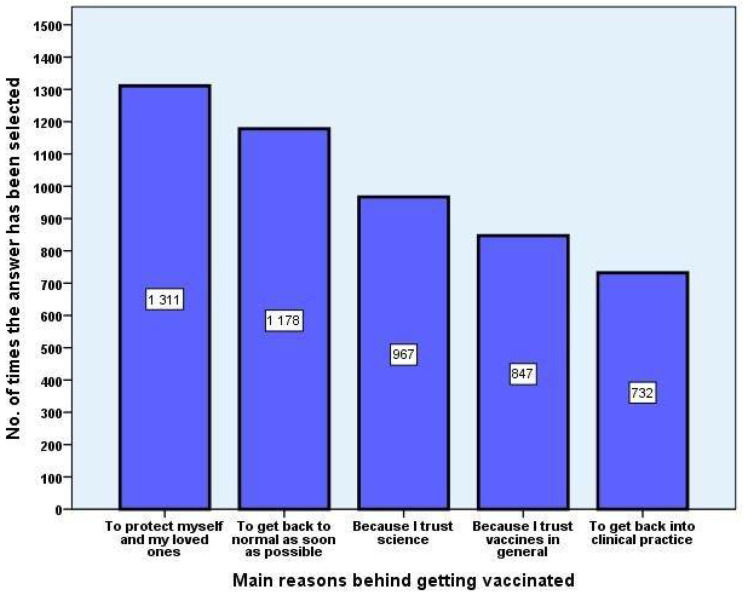
Main reasons for acceptance of COVID-19 vaccines. The top five most commonly chosen answers from a list of multiple pre-established ones were selected to visually represent the absolute frequency amongst replies.

The main five reasons for vaccination, choosing from a pre-established list with multiple-choice items, which allowed for open answers as well, were “To protect myself and my loved ones”, “To get back to normal as soon as possible”, “Because I trust science”, “I trust vaccines in general”, and “To get back into clinical practice” (Figure 1).

**Figure 2:**
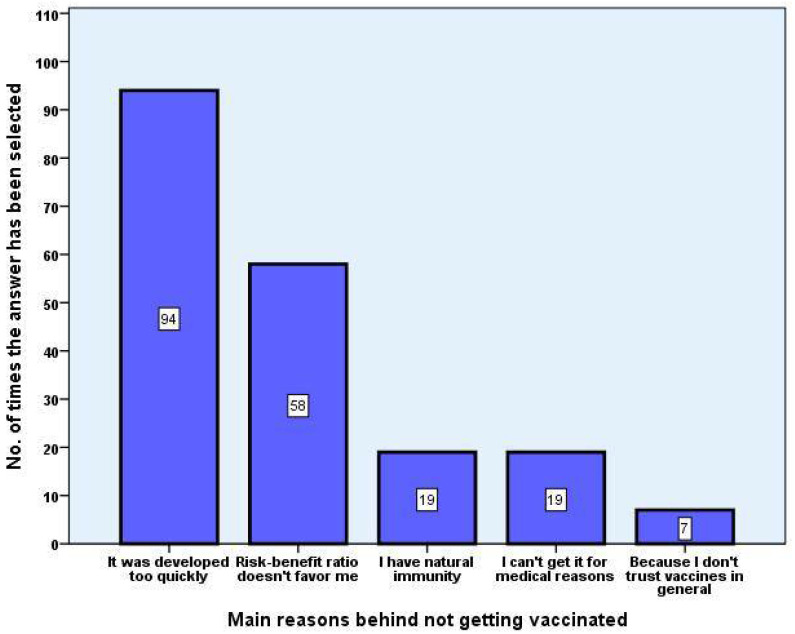
Main reasons for refusal of EU-approved COVID-19 vaccines. After being presented with several, the top five most frequent answers chosen from a pre-established list were visually generated as a bar plot to assess their frequency amongst replies.

When asked about their reasons for not vaccinating through a semi-structured multiple-choice questionnaire, the five main reasons were “It was developed too quickly”, “I cannot get it because of medical reasons”, “Risk-benefit ratio doesn’t favour me”, “I have natural immunity”, and “I do not trust vaccines in general” (Figure 2).

The main reason for vaccine rejection declared by healthcare students was related to the faster than usual development of vaccines, with strong agreement from the vaccine-resistant group and moderate agreement from the undecided group in (*p* < 0.001).

### 3.4. Perceived Knowledge on COVID-19 Vaccination

The majority of the respondents take their vaccine-related information from official and scientific sources. The national vaccination campaign is the most accessed information source (70.9%), followed by scientific journals (69.3%), university lecturers (62.8%), and the Ministry of Health page (53.5%). A moderate part of the respondents prefers news sites (21.9%). Friends and family represent a reliable source of information for only a modest part of the respondents (14.23% and 12.6%, respectively), while social media is the least popular source: Facebook (9.5%), TV (8.6%), public figures (7.3%), and Instagram (6.5%).

The perceived knowledge on vaccination correlates directly with the respondents’ attitude toward vaccination. Those included in the pro-vaccination group tend to consider themselves very well-informed, getting their information from trustworthy medical sources (34.2% in the pro-vaccination group vs. 22% in the vaccine resistant group and 19.5% in the undecided group, *p* < 0.001). Conversely, only 5% of the pro-vaccination group vs. 13.6% of the vaccine resistant and 17.9% of the undecided (*p* < 0.001) see themselves as poorly or very poorly informed. The majority (61.2%) of the respondents, irrespective of their vaccine stance, self-assessed as being very well or well-informed about the mRNA technology used by the vaccines approved in the EU at the moment of the study, 32.5% as having an intermediate amount of knowledge, and 5.3% as being poorly or very poorly informed.

### 3.5. Safety and Efficacy

The majority of the respondents (over 89%) trust the approved COVID-19 vaccines’ safety and efficacy. In a similar proportion, they agree that the available vaccines are efficient against symptomatic SARS-CoV-2 infection as well as against the severe form of the disease. They also agree that the available vaccines are useful for their individual protection, even though they do not place themselves in a high-risk category for a severe evolution of the infection.

The respondents self-assess as being well-informed on the vaccine-associated adverse reactions, correctly identifying the most frequent ones from a list of the officially reported side effects. Concern regarding the adverse reactions is present in 17.7% of respondents. Only 4.8% of respondents reported allergic reactions to vaccines in the past, while 32.6% know someone who has had allergic reactions to previous vaccines. Nevertheless, only 10.7% of them state that this fact affected their trust in vaccines.

The vaccination attitude correlates with concern over the development of adverse or allergic reactions (44.1% of those who are vaccine resistant and 26% of the undecided vs. 3.6% of those who are pro-vaccination, *p* < 0.001). Nevertheless, concern over the possible long-term effects of vaccines is present in only 11.5% of the overall respondents (52% of those undecided and 44.06% of the vaccine resistant vs. 5.4% of those who are pro-vaccination, *p* < 0.001).

### 3.6. Attitude towards Other Vaccines

The majority (76.3%) of the respondents confirm that they have received all the vaccines included in the national immunization programme, 17.4% do not know, and 6.3% are unvaccinated. Those unvaccinated represent 6.1% of the pro-vaccination group, 13.6% of the vaccine resistant, and 5.7% of the undecided group (*p* = 0.015).

Over 97% of the respondents consider vaccines included in the national immunization programme necessary. As future parents, 99.3% of those who are pro-vaccination, 95.1% of the undecided, and 89.1% of the vaccine resistant will vaccinate their children (*p* < 0.001). As medical professionals, 99.7% of those who are pro-vaccination, 93.5% of the undecided, and 89.8% of those who are vaccine resistant would advise parents to vaccinate their children (*p* < 0.001). In total, 90.7% of respondents would also encourage adults or children to get vaccinated with other vaccines not included in the national immunization programme (such as the HPV and flu vaccines), again with significant differences according to their attitude toward vaccination (93.4% of the pro-vaccination vs. 59.4% of the vaccine resistant and 74.8% of the undecided, *p* < 0.001). It is worth noting that even though the pro-vaccination students have a more positive attitude towards vaccination in general, only 32.6% of them received the flu vaccine in the 2020–2021 season. Nevertheless, this represents a significantly higher proportion than that in the vaccine-resistant or undecided group (10.2% and 13.8%, respectively, *p* < 0.001). Asked if the pandemic made them consider getting vaccinated against the flu, 59.8% of those pro-vaccination answered affirmatively vs. 12.5% of the vaccine-resistant respondents and 28.8% of the undecided.

### 3.7. Perception towards Vaccine Hesitancy in the General Population

**Figure 3:**
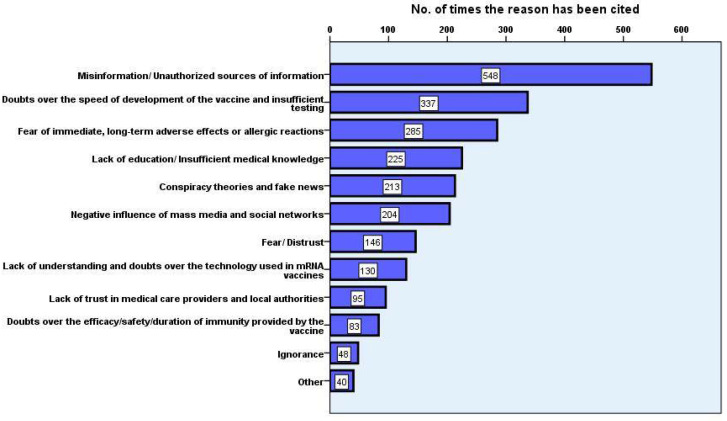
General themes regarding students’ perceptions of the general population’s reasons for COVID-19 vaccine hesitancy/refusal. Several themes have been identified as recurrent in the responders’ replies and chosen for their frequency to be visualised on the bar plot.

When asked to share their opinion about the main reasons for vaccine hesitancy within the general public, the interviewees’ answers mostly revolved around several themes, including misinformation, lack of medical education, negative impact of social media, and distrust in the local and medical authorities (Figure 3);

As per Table 2, various extracts from the respondents’ comments were reproduced and the smaller themes that were initially applied to group the answers were merged into two larger concepts so as to avoid the overlapping of topics generated by the complexity of open-ended responses. 

## 4. Discussion

In this study, undertaken during the first three months after approval of the first COVID-19 vaccines, the acceptance rate among Romanian healthcare students was quite high. Of all 1581 respondents, only a very small fraction (0.7%, 11 respondents) positioned themselves in absolute and indisputable disagreement with COVID-19 vaccination. Worldwide, similar pro-vaccination rates were reported: 91.9% in Poland [10] or 89.4% in India [11], while only 37.3% of medical students in Uganda were willing to get vaccinated [12]. A more nuanced attitude was present in medical students from Egypt, with 71% accepting vaccination but willing to postpone it—a likely undecided stance [13]. A study conducted on medical students in Michigan, US, revealed that despite high rates of vaccine acceptance, a quarter of medical students would refuse to get vaccinated immediately after FDA approval [14].

There is an undeniable discrepancy between the rates of vaccine acceptance in medical students and the general population. A survey performed in the general population in Romania revealed a much lower intention to get vaccinated: 55.2% [15]. Of those who are not willing to receive vaccination, 49% affirm that they could change their opinion, depending on further medical data regarding the adverse reactions and the vaccination rates of healthcare personnel. This underlines the important role played by medical professionals, students included, in overcoming vaccine hesitancy.

In a computer-assisted telephone interview conducted in the general population in Romania [16], lack of trust in the COVID-19 vaccine was the primary reason (45%) for vaccine hesitancy, while the respondents of the present study rate this as the least important reason (2.7%) in their decision-making process, placing a higher importance on the speed of vaccine development. This can shed light on the impact of different sources of information, as healthcare students tend to trust public health experts [11,14], while media exerts a higher influence on the general public’s attitude toward vaccination [17], acceptance of COVID-19 vaccine thus being associated with the ability to detect fake news [18].

An important point revealed by the present study was the overall positive attitude towards vaccination among healthcare students, irrespective of their perception of approved COVID-19 vaccines, with the nuances lying in how categorical their agreement was. Their present attitude will influence their behaviour as future parents and medical professionals, and, in the long term, could help shape a different perspective on vaccination in society. Rates of vaccine acceptance are expected to rise in the overall population if healthcare workers and local authorities get vaccinated [15]. Providing scientifically sound information about the personal and community-related benefits of vaccination [19] or adjusting the distributed data in a culturally sensitive manner can effectively educate underserved minorities [20]. Studies have shown that interventions as simple as addressing the fear of needles and injection may play an important role in curtailing vaccine reluctance [21].

This study has several limitations. The high percentage of students with a pro-vaccination attitude in the present study can signal a response bias, since the total number of respondents represent only 14.9% of the entire student population of Carol Davila University of Medicine. However, 70% of the students in the University signed up for vaccination at the beginning of the actual campaign in January 2021, and the questionnaire was open until the third of March, thus offering two months for reconsideration of their vaccine attitude. Moreover, the sample representativeness is further confirmed by the fact that by the time of manuscript’s submission, in June 2021, over 80% of the total number of students in the Carol Davila University of Medicine and Pharmacy had already been vaccinated.

Another limitation is the used Likert scale, which leaves room for a subjective over-evaluation of the self-perceived degree of comprehension and knowledge. Moreover, it is to be stated that both the pro-vaccination and the vaccine-resistant students may have been inclined to adjust their responses in order to reinforce their attitude toward vaccination.

An assessment of the impact of medical literacy on vaccination attitudes in non-healthcare students and the general population represents a future research direction, together with a perspective on COVID-19 vaccines that were further approved in the European Union.

## 5. Conclusions

Healthcare students, proving to have an overall positive attitude towards vaccination, can become a crucial resource in spreading essential, scientifically sound information to the general public. The toll of COVID-19 has weighed down and will continue to do so heavily on the healthcare system as long as the vaccination rate in the general population remains low. Enthusiastic and dynamic students, acting as volunteers, can increase public trust in medical professionals, the healthcare system, and authorities, thus diminishing vaccine hesitancy. In order to achieve this, they can use innovative and creative communication through user-friendly social media campaigns, reaching above and beyond the youth sector. Their willingness and solidarity can be used to promote non-conventional vaccination campaigns such as marathons and drive-through points open to the general population, which would be a substantial contribution to reaching the target level of herd immunity.

## Figures and Tables

**Table 1:** Factors associated with vaccine acceptance in the respondent population comprising medical students.

Factors	Overall Respondents (*n* = 1581)		Pro-Vaccine Group (*n* = 1399)		Undecided Group (*n* = 123)		Vaccine Resistant Group (*n* = 59)		*p*-Value
	***n***	**%**	***n***	**%**	***n***	**%**	***n***	**%**	
Faculty									
General Medicine	1146	72,5%	1053	75.3%	64	52.0%	29	49.2%	<0.001
Dentistry	216	13.7%	177	12.7%	27	22.0%	12	20.3%	
Pharmacy	175	11.1%	135	9.6%	25	20.3%	15	25.4%	
Midwifery and Nursery	44	2.8%	34	2.4%	7	5.7%	3	5.1%	
Trust efficacy of COVID-19 vaccines									
Yes	1460	92.3%	1366	97.6%	74	60.2%	20	33.9%	<0.001
No	121	7.7%	33	2.4%	49	39.8%	39	66.1%	
Trust safety of COVID-19 vaccines									
Yes	1414	89.4%	1350	96.5%	50	40.7%	14	23.7%	<0.001
No	167	10.6%	49	3.5%	73	59.3%	45	76.3%	
Opinion: COVID-19 vaccines were developed too fast									
Strongly disagree	383	24.2%	377	26.9%	4	3.3%	2	3.4%	<0.001
Disagree	477	30.2%	463	33.1%	11	8.9%	3	5.1%	
Neither agree nor disagree	397	25.1%	358	25.6%	32	26.0%	7	11.9%	
Agree	166	10.5%	128	9.1%	28	22.8%	10	16.9%	
Strongly agree	158	10.0%	73	5.2%	48	39.0%	37	62.7%	
mRNA knowledge self-assessment									
A lot and can easily explain to others	515	32.6%	478	34.2%	24	19.5%	13	22.0%	<0.001
A lot, but find it hard to explain to others	452	28.6%	409	29.2%	29	23.6%	14	23.7%	
Intermediate amount of knowledge	514	32.5%	442	31.6%	48	39.0%	24	40.7%	
Little knowledge on the subject	78	4.9%	55	3.9%	16	13.0%	7	11.9%	
Too little or no knowledge at all	22	1.4%	15	1.1%	6	4.9%	1	1.7%	
Concern over possible allergic or adverse reaction									
Strongly disagree	441	27.9%	425	30.4%	12	9.8%	4	6.8%	<0.001
Disagree	492	31.1%	462	33.0%	21	17.1%	9	15.3%	
Neither agree nor disagree	377	23.8%	332	23.7%	33	26.8%	12	20.3%	
Agree	163	10.3%	130	9.3%	25	20.3%	8	13.6%	
Strongly agree	108	6.8%	50	3.6%	32	26.0%	26	44.1%	
Concern over possible long-term adverse reaction									
Strongly disagree	453	28.7%	447	32.0%	5	4.1%	1	1.7%	<0.001
Disagree	425	26.9%	413	29.5%	9	7.3%	3	5.1%	
Neither agree nor disagree	330	20.9%	313	22.4%	13	10.6%	4	6.8%	
Agree	191	12.1%	150	10.7%	32	26.0%	9	15.3%	
Strongly agree	182	11.5%	76	5.4%	64	52.0%	42	71.2%	
Full immunization as per national scheme									
Yes	1206	76.3%	1084	77.5%	85	69.1%	37	62.7%	0.015
No	100	6.3%	85	6.1%	7	5.7%	8	13.6%	
I don′t know	275	17.4%	230	16.4%	31	25.2%	14	23.7%	
Opinion: it is necessary to be immunized as per national scheme									
Yes	1539	97.3%	1372	98.1%	115	93.5%	52	88.1%	<0.001
No	42	2.7%	27	1.9%	8	6.5%	7	11.9%	
As future parent: vaccination acceptance									
Yes	1559	98.6%	1389	99.3%	117	95.1%	53	89.8%	<0.001
No	22	1.4%	10	0.7%	6	4.9%	6	10.2%	
As future medical care provider: vaccination recommendation									
Yes	1563	98.9%	1395	99.7%	115	93.5%	53	89.8%	<0.001
No	18	1.1%	4	0.3%	8	6.5%	6	10.2%	
Vaccinated against the flu this season?									
Yes	479	30.3%	456	32.6%	17	13.8%	6	10.2%	<0.001
No/Not yet	1102	69.7%	943	67.4%	106	86.2%	53	89.8%	

**Table 2:** Cited opinions regarding the general population’s reasons for refusing EU-approved COVID-19 vaccines. Short extracts from the answers provided to the question “What do you think the main reasons behind vaccine hesitancy in the general population are?”, grouped according to relevant general theme.

General Theme	Cited Opinion
Information and knowledge	“[…] not even the pro-vaccine side can be efficient in informing the greater public, if they do not adjust the information to the average level of understanding.”
	“The anti-science propaganda started from the misunderstanding, or lack of understanding, regarding notions of basic biology and physiology.”
	“Many public figures without any medical training have stepped forward on social media and spread their opinions containing wrong or misunderstood medical information, thus confusing the public.”
	“There is a general ignorance regarding health issues among common people, “If it does not hurt, it does not need to be treated.”
	“(…) due to unauthorized sources of information, people have doubts over the content of the vaccine itself, some unreliable media outlets even suggesting they might contain foreign tracking devices, while at the same time placing emphasis on trusting plant-based medicine.”
Emotional response	“(…) the feeling of being overwhelmed by such a massive event—a pandemic—lead to people using reluctance as a coping mechanism.”
	“I have felt personally affected by articles with fake information or conspiracy theories; however illogical they may be, it suffices if they allow for the feeling of restlessness and mistrust to collectively appear.”
	“As far as the long-term adverse reaction theories go, there is still a number of people who believe and fear that the vaccine is the source of future neoplasms or fertilty issues.”
	“The reluctance comes from the fact that there are insufficient studies proving long-term efficiency of the vaccine; therefore, people believe they are going to need boosters every three months or so.”
	“There are still doubts regarding whether the vaccine lowers the transmisibility of the virus, when the vaccinated person is infected or exposed.”
	“(…) the fact that most people feel disappointed by our national medical system and thus easily reach the conclusion that the national immunization campaign is driven by other purposes than simply vaccinating men and women.”
	“People believe that the vaccine is still being tested on the general population, due to its hasty development.”

## Data Availability

All data presented are available upon request from corresponding author (A.B.).

## Appendix A. Questionnaire

GDPR Statement

A. General Data

Gender (Male/Female/Prefer not to answer)

Age

Citizenship

Background (Urban/Rural)

Faculty (Medicine, Dentistry, Pharmacy, Nurses and Midwives)

B. COVID19 Infection and vaccination

Have you tested positive for COVID19? (Yes/No)

If yes, by which type of test? (RT-PCR/rapid antigen test/rapid antibody test)

If yes, what was the course of the disease? (asymptomatic/mild symptoms/moderate symptoms/severe symptoms)

Do you suffer from a chronic illness? (oncologic, cardiovascular, chronic pulmonary disease, obesity, or diabetes) (Yes/No)

Will you get vaccinated against COVID19? (I am already vaccinated/I will get vaccinated/No/Not yet, I am waiting for my colleagues to get it first/Maybe)

If you will get vaccinated, why? (Semi-structured, multiple-choice question)

If you will not get vaccinated, why? (Semi-structured, multiple-choice question)

If you will not get vaccinated, what would convince you? (Open-ended question)

Do you think that healthcare students have a higher risk of infection than the general population? (Yes/No)

C. Perception on safety and efficacy

Do you trust the efficacy of EU-approved anti-COVID19 vaccines? (Yes/No)

Do you trust the safety of EU-approved anti-COVID19 vaccines? (Yes/No)

The vaccine prepares the immune system to fight against the symptomatic infection with SARS-CoV-2: (Likert scale)

The vaccine helps avoid the severe disease, in case of infection: (Likert scale)

The vaccine is useful even though I do not belong to a risk category: (Likert scale)

The vaccine was developed too quickly: (Likert scale)

What do you believe is the main reason for vaccine hesitancy in the general population? (Open-ended question)

D. Perception of knowledge on COVID-19 vaccines

How many things do you know about the mARN technology used in the EU approved COVID-19 vaccines? (Nothing or very few things/Few things/Average/Many things, but I cannot easily explain/Very much and I can easily explain to others)

Which of the following technologies used in vaccine development do you trust more, in regard to safety and efficacy? (mARN (Pfizer and Biontech/Moderna)/adenovirus vector (Oxford and AstraZeneca, Janssen)/recombinant protein (Sanofi/GSK)/inactivated virus (Valneva)/others)

Which sources do you get your information from? (12 pre-established answers and others)

E. Perception regarding adverse and/or allergic reactions

What are the possible adverse reactions associated with the mARN technology vaccines? (9 pre-established answers and others)

I am worried I will develop an adverse or allergic reaction to the vaccine: (Likert scale)

I am worried about the long-term adverse reactions: (Likert scale)

Did you have allergic reactions to other vaccines? (Yes/No)

Do you know someone who has ever had an allergic reaction to any vaccine? (Yes/No)

If yes, did it affect your trust in vaccines? (Yes/No)

F. Perception regarding other vaccines

Were the vaccines included in the national vaccination program administered to you? (Yes/No/I do not know)

Do you believe it is necessary that all children receive the vaccines in the national vaccination program?) (Yes/No)

As a future parent, will you have your children vaccinated with the vaccines included in the vaccination program? (Yes/No)

As a future doctor, would you recommend vaccines that are not included in the vaccination program, but proved effective? (Yes/No)

I believe that the vaccine against HBV is effective and safe: (Likert scale)

I believe that the vaccine against HPV is effective and safe: (Likert scale)

I believe that the vaccine against the flu is effective and safe: (Likert scale)

I believe it is necessary to get vaccinated against: (HBV/HPV/flu)

Have you gotten the flu vaccine this season? (Yes/No/Not yet)

Did the COVID19 pandemic make you consider more getting the flu vaccine? (Yes/No)

I believe that optional vaccines and booster shots needed for travelling are useful: (Likert scale)

## References

[1] Shimizu Y., WHO (2019). Vaccines and Immunization. https://www.who.int/health-topics/vaccines-and-immunization#tab=tab_1.

[2] Popescu C.P., Marin A., Melinte V., Gherlan G.S., Banicioiu F.C., Dogaru A., Smadu S., Veja A.M., Nedu E., Stanciu D. (2020). COVID-19 in a tertiary hospital from Romania: Epidemiology, preparedness and clinical challenges. Travel Med. Infect. Dis..

[3] CNSCBT-COVID-19 Weekly Surveillance Report—Data Reported until 18th of July. https://www.cnscbt.ro/index.php/analiza-cazuri-confirmate-covid19/2585-raport-saptamanal-episaptamana28-2021/file.

[4] (2020). European Medicines Agency Comirnaty EU Product Information. https://www.ema.europa.eu/en/documents/product-information/comirnaty-epar-product-information_ro.pdf.

[5] National Coordinating Committee for Vaccination Programme against COVID-19-Report of Number of People Vaccinated Against COVID-19 on 20th of July. https://vaccinare-covid.gov.ro/actualizare-zilnica-20-07-evidenta-persoanelor-vaccinate-impotriva-covid-19/.

[6] CNSCBT-COVID-19-Assessment of the Current National Situation and Future Outlook. https://www.cnscbt.ro/index.php/analiza-cazuri-confirmate-covid19/2584-evaluare-situatie-si-perspectiva-14-07-2021/file.

[7] Gallup. Wellcome Global Monitor—How Does the World Feel about Science and Health, 2018. https://wellcome.org/sites/default/files/wellcome-global-monitor-2018.pdf.

[8] WHO/R Akbar Ten Threats to Global Health in 2019. https://www.who.int/news-room/spotlight/ten-threats-to-global-health-in-2019.

[9] Australian Bureau of Statistics—Sample Size Calculator. https://www.abs.gov.au/websitedbs/d3310114.nsf/home/sample+size+calculator.

[10] Szmyd B., Bartoszek A., Karuga F.F., Staniecka K., Błaszczyk M., Radek M. (2021). Medical Students and SARS-CoV-2 Vaccination: Attitude and Behaviors. Vaccines.

[11] Jain J., Saurabh S., Kumar P., Verma M., Goel A., Gupta M., Bhardwaj P., Raghav P. (2021). COVID-19 vaccine hesitancy among medical students in India. Epidemiol. Infect..

[12] Kanyike A.M., Olum R., Kajjimu J., Ojilong D., Akech G.M., Nassozi D.R., Agira D., Wamala N.K., Asiimwe A., Matovu D. (2021). Acceptance of the coronavirus disease-2019 vaccine among medical students in Uganda. Trop. Med. Health.

[13] Saied S.M., Saied E.M., Kabbash I.A., Abdo S.A.E. (2021). Vaccine hesitancy: Beliefs and barriers associated with COVID-19 vaccination among Egyptian medical students. J. Med. Virol..

[14] Lucia V.C., Kelekar A., Afonso N.M. (2020). COVID-19 vaccine hesitancy among medical students. J. Public Health.

[15] INSCOP Research—Public Perception over Romanians’ Trust in COVID-19 Vaccination. https://www.inscop.ro/08-februarie-2021-perceptia-publica-asupra-vaccinarii-increderea-romanilor-in-vaccinarea-anti-covid19-capitolul-i/.

[16] Statista J.A. Most Important Reasons for Refusing the Vaccine against COVID-19 in Romania 2020. https://www.statista.com/statistics/1195987/romania-reasons-for-refusing-the-vaccine-against-covid-19/.

[17] Miko D., Costache C., Colosi H.A., Neculicioiu V., Colosi I.A. (2019). Qualitative Assessment of Vaccine Hesitancy in Romania. Medicina.

[18] Montagni I., Ouazzani-Touhami K., Mebarki A., Texier N., Schück S., Tzourio C. (2021). The CONFINS group, Acceptance of a Covid-19 vaccine is associated with ability to detect fake news and health literacy. J. Public Health.

[19] Freeman D., Loe B.S., Yu L.M., Freeman J., Chadwick A., Vaccari C., Shanyinde M., Harris V., Waite F., Rosebrock L. (2021). Effects of different types of written vaccination information on COVID-19 vaccine hesitancy in the UK (OCEANS-III): A single-blind, parallel-group, randomised controlled trial. Lancet Public Health.

[20] Hildreth J., Alcendor D.J. (2021). Targeting COVID-19 Vaccine Hesitancy in Minority Populations in the US: Implications for Herd Immunity. Vaccines.

[21] Freeman D., Lambe S., Yu L., Freeman J., Chadwick A., Vaccari C., Waite F., Rosebrock L., Petit A., Vanderslott S. (2021). Injection fears and COVID-19 vaccine hesitancy. Psychol. Med..

